# Temperature-responsive hydrogel of oclacitinib maleate administered via the rectum in rabbit

**DOI:** 10.3389/fvets.2026.1767033

**Published:** 2026-02-11

**Authors:** PanPan Guo, Yang Yang, Zhongsheng Zhang, Yuxin Guo, Muhammad Mohsin, Haiyan Wu, Dengfeng Wang, Guangwen Yin, Lei Wang

**Affiliations:** 1 College of Animal Sciences, Fujian Agriculture and Forestry University, Fuzhou, China; 2 Academy of Integrative Medicine, Fujian University of Traditional Chinese Medicine, Fuzhou, China

**Keywords:** hydrogel, oclacitinib maleate, pharmacokinetics, rectal administration, temperature-responsive

## Abstract

**Background:**

The extensive and intense itching caused by skin diseases can significantly diminish the quality of life for companion animals. Oral administration of oclacitinib maleate is recognized as an important treatment for alleviating itching. Oral medications are affected by the animal’s temperament and swallowing ability.

**Methods:**

In this study, we developed a temperature-responsive hydrogel containing oclacitinib maleate for rectal administration, and we evaluated its therapeutic potential for treating itching through pharmacokinetic analysis using rabbits as test subjects.

**Results:**

Pharmacokinetic results indicated that the transrectal administration of OM T-R Hydrogel effectively increased the blood concentration of oclacitinib maleate (T_max_ = 87 min, C_max_ = 37 ng/mL, t_1/2λz_ = 762 min). Furthermore, the OM T-R Hydrogel demonstrated no rectal mucosal irritation, confirming its safety for clinical use.

**Conclusion:**

In conclusion, this paper successfully investigated a promising novel drug delivery system to alleviate pruritus in dogs and cats.

## 1 Introduction

Skin diseases are among the most prevalent clinical conditions observed in pets. The etiology of these diseases is complex and includes factors such as dermatophytes, parasites, and autoimmune responses (1–3). Pruritus is one of the most common symptoms associated with dermatological conditions in pets, and widespread, intense itching significantly impacts their overall quality of life (4, 5). Therefore, pruritus management is crucial for ensuring animal welfare. In the pet clinic, the use of anti-itch medications is the most common method of controlling itching (6). Oclacitinib maleate (OM, Apoquel®), a Janus kinase (JAK) inhibitor, has demonstrated efficacy in alleviating itching associated with various allergies, including flea, food, and contact allergies, by down-regulating the expression of several inflammatory cytokines linked to JAK 1 (7–9). In veterinary practice, oral administration of OM is commonly employed due to its simplicity and stability as a drug delivery method (10, 11). Frequent oral dosing of OM in pets with severe itching creates notable obstacles related to convenience and ease of administration (12). However, it is essential to consider that the temperament and swallowing abilities of dogs and cats are significant factors influencing the success of oral administration (13). Specifically, irritable or sensitive pets may resist oral medication and spit out the drug, while those with oral or esophageal diseases, or neurological disorders that impair the swallowing reflex, are also unable to receive adequate medication via the oral route (14, 15). On the other hand, in cases involving irritable animals, the risk of bites to the veterinarian increases substantially (16). As a result, rectal administration has emerged as a viable alternative for drug delivery that warrants attention from veterinarians (17–19).

In contrast to humans, animals lack conscious control of the external anal sphincter during rectal drug delivery, leading to a higher chance of medication leakage. Temperature-Responsive Hydrogel (T-R Hydrogel) is a specialized type of hydrogel that can alter its physical state in response to temperature stimuli (20). Due to its unique physicochemical properties, the T-R hydrogel finds extensive applications in the field of biomaterials (21–23). In this study, we developed an Oclacitinib Maleate T-R Hydrogel (OM T-R Hydrogel), which exists as a liquid at room temperature and transitions to a semi-solid state at a phase change temperature of 38 °C. Our objective was to leverage the bioadhesive properties of semi-solid hydrogels to minimize drug leakage during rectal administration. To further investigate the feasibility of OM T-R Hydrogel for clinical use in veterinary medicine, we conducted an exploratory study utilizing rabbits as experimental animal. Following the injection of OM T-R Hydrogel into the rectum via a catheter, venous blood samples were collected at various time intervals. Moreover, the concentration of OM in rabbits was determined by liquid chromatography-mass spectrometry.

## 2 Materials and methods

### 2.1 Animals and reagents

Rabbits and mice were obtained from the Meat Rabbit Farm of Fujian Agriculture and Forestry University and HFK Bio-Technology Co., Ltd. (Beijing, China), respectively, and the animal experiment was approved by the Ethics Committee of Fujian Agriculture and Forestry University (FAFU-2023-0065). Oclacitinib Maleate, Poloxamer 407 (P407), Poloxamer 188 (P188), Hydroxypropyl methyl cellulose (HPMC), Rhodamine B were purchased from Beijing Psaitong Biotechnology Co., Ltd. (Beijing, China). Hydroxypropyl-*β*-Cyclodextrin (HP-β-CD) was purchased from Sangon Biotechnology Co., Ltd. (Shanghai, China).

### 2.2 Preparation and characterization of OM T-R hydrogel

A total of 30 mg of OM was accurately weighed and dissolved in 30 mL of ultrapure water to prepare an aqueous solution of OM at a concentration of 1 mg/mL. Subsequently, 0.132 g (0.44%, w/v) of HPMC was weighed as a mucosal adhesive, and 0.75 g (2.5%, w/v) of HP-*β*-CD was weighed as an absorption enhancer; both were thoroughly dissolved in the aqueous OM solution (24, 25). Following this, 5.4 g (18%, w/v) of P407 and 1.2 g (4%, w/v) of P188 were added. The mixture was refrigerated at 4 °C for 24 h to ensure complete dissolution, resulting in the formation of the OM hydrogel.

The prepared T-R Hydrogel and OM T-R Hydrogel were lyophilized separately for 48 h. Subsequently, they were subjected to further observation using scanning electron microscopy and Fourier transform infrared spectroscopy. Additionally, we conducted temperature responsiveness validation of the OM T-R Hydrogel, which included both *in vitro* and *in vivo* assessments. The in vitro temperature responsiveness validation was executed by establishing a temperature gradient of 37 °C to 42 °C using a water bath, with three batches undergoing multiple repetitions. The *in vivo* temperature responsiveness validation involved the injection of rhodamine B, dissolved in the OM T-R Hydrogel, into the rectum of rabbits, followed by observation for any leakage within a 10 min period.

### 2.3 Animal experiment

Prior to the commencement of the animal experiments, we collected 2 mL of venous blood for backup purposes. Subsequently, eight rabbits were weighed and randomly assigned to two groups: the oral drug group (O group, *n* = 3) and the rectal drug group (R group, *n* = 5). The OM was administered at a dosage of 1 mg/kg. Prior to drug administration, the rabbits were fasted for 18 h, and it was confirmed via X-ray that no fecal residue was present in the colonic and rectal segments. Following drug administration, a specific volume of air was injected into the drug delivery tube using a syringe to expel the drug as thoroughly as possible.

At 25 min, 50 min, 1.5 h, 3 h, 6 h, 8 h, 10 h, and 12 h post-drug administration, we collected venous blood into EDTA-K_2_ anticoagulation tubes. After centrifugation at 3500 g for 10 min at 4 °C, 50 μL of plasma was separated into polyethylene tubes. Protein precipitation was achieved by adding 100 μL of ice-cold acetonitrile at −20 °C. The samples were vortexed for 5 min and subsequently centrifuged at 20000 g for 10 min at 4 °C. Following centrifugation, the supernatant was transferred to a conical polypropylene tube designed for liquid chromatography-mass spectrometry (LC–MS), which was then capped and placed in an autosampler for injection.

### 2.4 Establishment of relevant parameters of liquid chromatography-mass spectrometry and standard curves

This method was established based on the study conducted by Ferrer et al. (26) The chromatographic column used was a ThermoFisher Accucore C18 (2.6 μm, 4.1 × 150 mm) with a column temperature of 40 °C and an autosampler set at 8 °C. The mobile phase flow rate was maintained at 0.8 mL/min and consisted of a gradient mixture of acetonitrile and water, both containing 0.1% formic acid, in the following ratios: the initial condition was 5% acetonitrile and 95% water. After the injection of 4 μL, the ratio changed linearly to 60% acetonitrile within 4 min, then increased to 90% acetonitrile within 0.1 min, which was held for 6 min. Finally, it was returned to the initial condition at 6.1 min and held for 1.9 min. Under these conditions, the retention time of OM was 3.62 min. The assay was conducted using positive electrospray ionization (ESI+), and the quantitative analysis was monitored for transitions from 338.2 to 149.2 atomic mass units (amu).

A precisely weighed 12 mg of OM was dissolved in 10 mL of distilled water to prepare a stock solution. Subsequently, 1 mL of this stock solution was diluted to a final volume of 100 mL with distilled water to obtain the working solution. A multiple dilution method was employed to prepare various concentrations of the drug solution. Add 50 μL of blank serum to each of the different concentrations of drug solutions. Mix thoroughly to prepare 10 concentration gradients of standard solutions: 2000 ng/mL, 1,000 ng/mL, 500 ng/mL, 250 ng/mL, 125 ng/mL, 62.5 ng/mL, 31.25 ng/mL, 15.625 ng/mL, 7.8125 ng/mL, and 3.905 ng/mL. Subsequently, the standard solutions were prepared according to the procedure outlined in section 2.3 for injection. Considering the specific conditions of this test, a concentration range of 250 ng/mL to 3.905 ng/mL was ultimately selected to establish a standard curve, which included a total of seven concentration points.

### 2.5 Acute toxicity test of OM T-R hydrogel

For seven consecutive days, 0.5 mL of OM T-R Hydrogel was withdrawn and administered rectally in mice, during which spillage of the hydrogel from the anus was observed. Tissue sections from the anal end of the rectum were prepared to histologically assess the irritation caused by OM T-R Hydrogel at the site of administration. Additionally, serum samples were collected to measure ALT, AST, CREA, BUN, TBIL, and ALP to evaluate the hepatic and renal effects of OM T-R Hydrogel.

### 2.6 Statistical analysis

Inter-group differences were assessed using one-way analysis of variance (ANOVA) by Dunnett’s test. The experimental data presented in this study are expressed as the mean ± standard deviation (SD) derived from a minimum of three independent experiments. Statistical significance was defined as *p < 0.05, **p < 0.01. The data were analyzed and processed using Phoenix, Excel, and Origin 2024 software.

## 3 Results

### 3.1 Characterization of OM T-R hydrogel

**Figure 1:**
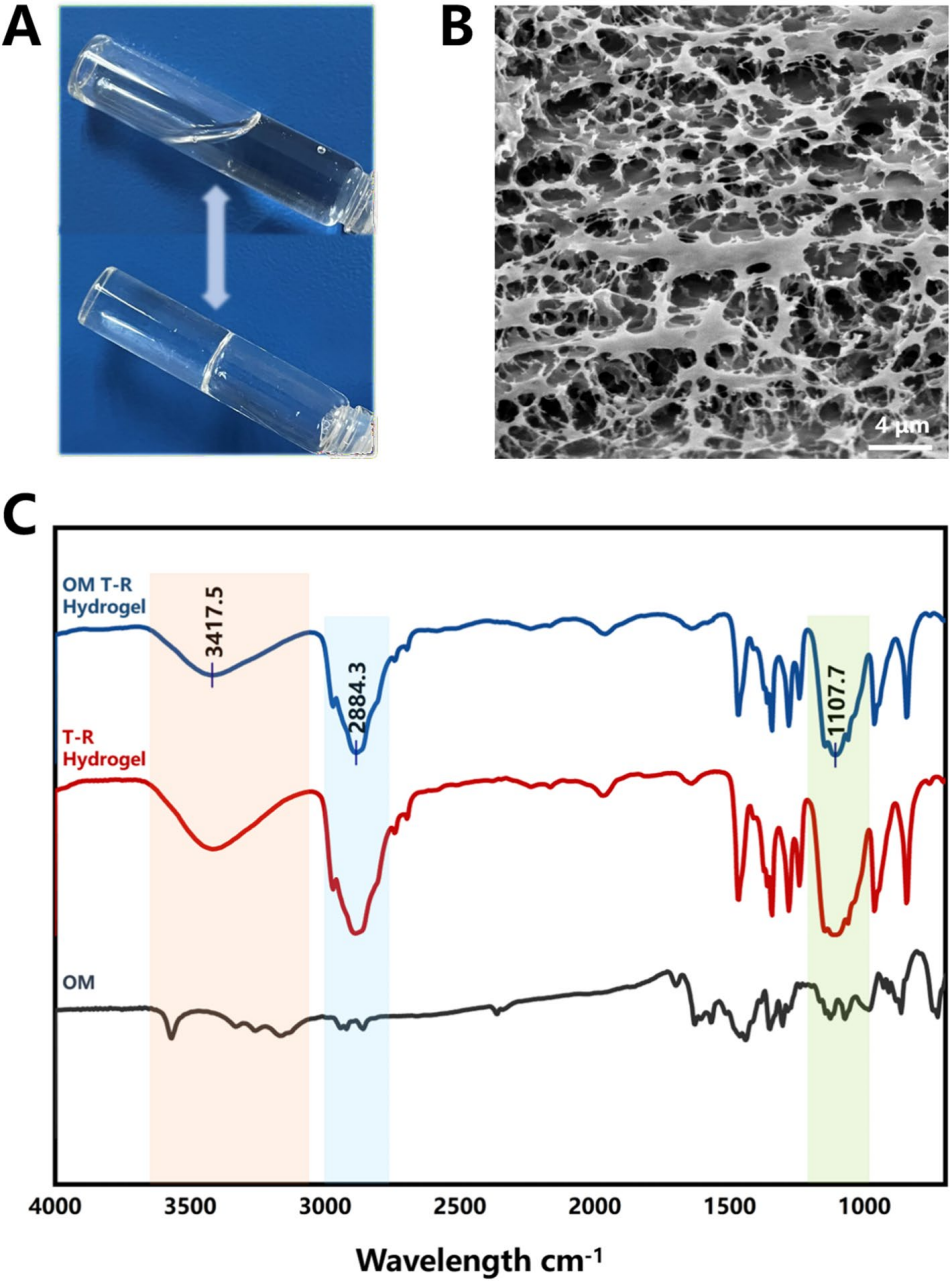
Characterization of OM T-R hydrogel. **(A)** Temperature responsiveness of OM T-R hydrogel; **(B)** SEM of OM T-R hydrogel; **(C)** FTIR analysis.

At room temperature, the OM T-R Hydrogel exhibited a uniform texture and good flowability (Figure 1A). The transformation of the T-R Hydrogel into a semi-solid state was successfully achieved by maintaining it at 38 °C for 30 s. However, the OM T-R Hydrogel required 39 s at 38 °C to effectively accomplish the phase transition (Supplementary data). The results from the *in vivo* temperature responsiveness test indicated that the rabbits’ anal region was dry, and the surrounding fur maintained a normal color without any signs of leakage, based on the condition that the rabbits had not defecated. Scanning electron microscopy (SEM) images revealed that T-R Hydrogel possesses a three-dimensional mesh architecture characterized by numerous pores and channels of varying sizes (Figure 1B), which is advantageous for drug loading and diffusion. Fourier Transform Infrared Spectroscopy (FTIR) results indicated that both OM T-R Hydrogel and T-R Hydrogel exhibited characteristic absorption bands corresponding to -OH stretching vibrations (3200–3,600 cm^−1^), C-O stretching vibrations (1000–1,250 cm^−1^), C-H stretching vibrations (2800–3,000 cm^−1^), and bound water peaks. Furthermore, the wavelengths of the other absorption peaks for both hydrogels were found to be consistent (Figure 1C). This observation suggests that T-R Hydrogel does not chemically react with OM to form new chemical bonds, implying that T-R Hydrogel functions solely as a delivery system.

### 3.2 Pharmacokinetics

This exploratory study was conducted in 8 healthy rabbits. Prior to the study, clinical physical examination revealed no significant abnormalities. All rabbits showed no adverse effects at the end of the animal experiment.

**Figure 2:**
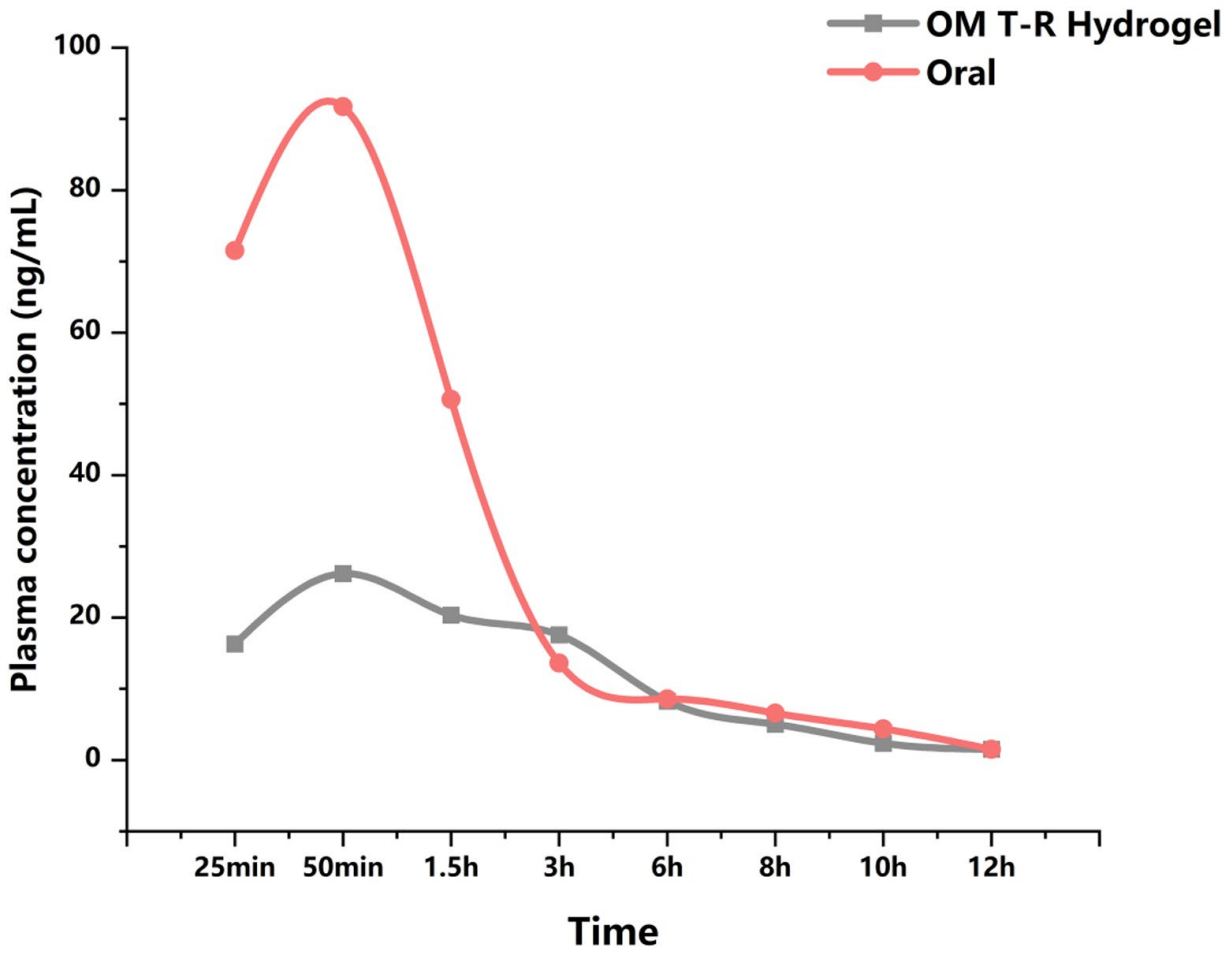
Plasma O-M concentration-time curve.

Table 1 shows the changes in the plasma concentrations (ng/mL) of OM over time in 8 rabbits under oral administration (*n* = 3) and rectal administration (*n* = 5), respectively. The changes in the average concentrations over time for the two groups are shown in Figure 2. The main pharmacokinetic data for oral and rectal administration are presented in Table 2. The results indicate that for the oral administration group (Group O), the T_max_ was 42 min, the C_max_ was 94 ng/mL, the t_1/2λz_ was 522 min, and the Cl-F was 163 mL/min; for the rectal administration group (Group R), the T_max_ was 87 min, the C_max_ was 37 ng/mL, the t_1/2λz_ was 762 min, and the Cl-F was 316 mL/min.

**Table 1:** Plasma O-M concentrations (ng/mL) over time in eight rabbits following treatment.

Time\Animal ID	25 min	50 min	1.5 h	3 h	6 h	8 h	10 h	12 h
Rabbit A1	27.00	79.21	42.32	15.87	7.48	8.73	7.16	1.53
Rabbit A2	90.56	105.04	60.27	11.25	5.91	4.98	2.57	1.50
Rabbit A3	96.98	90.88	49.32	13.75	12.40	6.09	3.45	1.54
Mean	71.51	91.71	50.64	13.63	8.59	6.60	4.39	1.52
SD	31.58	10.56	7.39	1.89	2.76	1.57	1.99	0.01
Rabbit B1	33.09	17.84	8.46	5.91	4.70	3.25	2.77	1.97
Rabbit B2	18.05	45.09	7.22	19.02	8.57	5.77	2.18	1.48
Rabbit B3	8.56	15.06	23.36	10.69	6.21	4.53	2.33	1.34
Rabbit B4	12.36	41.84	51.70	21.03	13.95	6.44	2.17	1.45
Rabbit B5	9.39	10.97	11.08	31.26	7.94	5.23	2.38	1.40
Mean	16.29	26.16	20.36	17.58	8.27	5.04	2.37	1.53
SD	9.04	14.33	16.68	8.77	3.14	1.10	0.22	0.23

Rabbit A1-3 using oral administration; Rabbit B1-5 using rectal administration.

**Table 2:** Pharmacokinetic parameters of oral (*n* = 3) and rectal (*n* = 5) administration.

Pharmacokinetic parameter	Oral administration Mean ± Standard	Rectum administration Mean ± Standard
Dose (mg/kg)	1	1
T_max_ (min)	41.67 ± 11.79	87.00 ± 52.69
C_max_ (ng/mL)	93.74 ± 10.79	36.90 ± 10.15
t_1/2λz_ (min)	521.89 ± 100.13	761.71 ± 356.57
AUC_0-last_ (min*ng/mL)	13957.03 ± 566.44	8267.64 ± 2106.43
AUC_0-inf_ (min*ng/mL)	15103.53 ± 727.39	10061.81 ± 1765.75
MRT_last_ (min)	284.95 ± 51.95	378.73 ± 53.32
MRT_0-inf_ (min)	431.36 ± 16.44	752.35 ± 518.92
Cl-F (mL/min)	163.50 ± 7.47	315.70 ± 154.60

T_max_ indicates the time to reach maximum plasma concentration; C_max_ indicates the maximum plasma concentration; t_1/2λz_ indicates the terminal elimination half-life; Cl-F indicates the clearance divided by absolute bioavailability.

### 3.3 Hepatorenal toxicity and rectal mucosal irritation associated with OM T-R hydrogel

**Figure 3:**
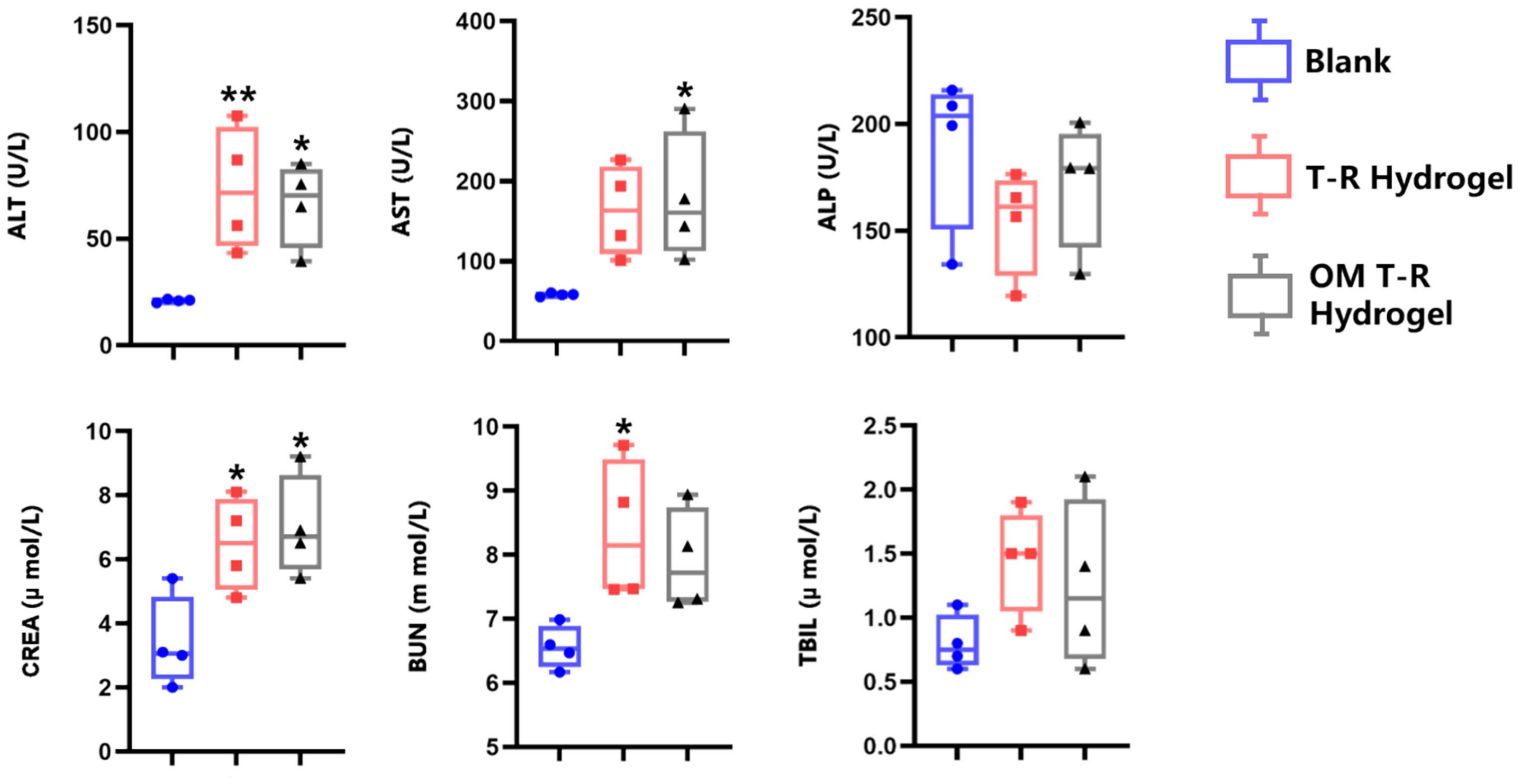
Serum biochemical indices in different experimental groups. A comparison is conducted between the experimental group and the blank group. *p < 0.05; **p < 0.01.

**Figure 4:**
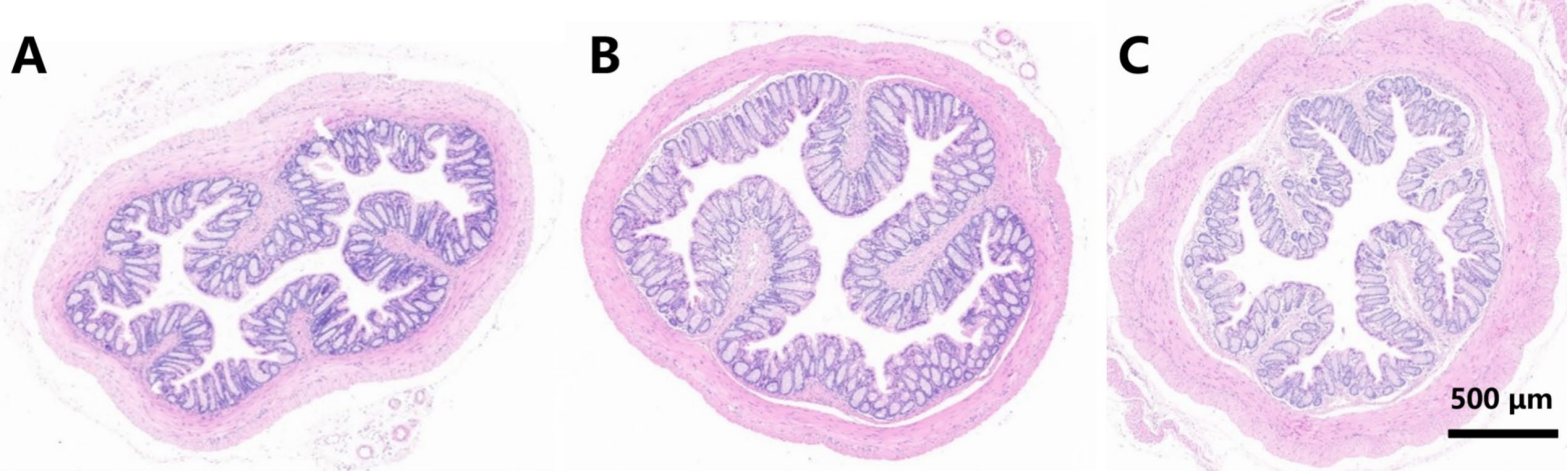
Histological section of the rectum (anal end) tissue. **(A)** Blank group; **(B)** treatment with 0.9% NaCl; **(C)** treatment with OM T-R hydrogel.

After the experiment, there were no significant abnormalities observed in the mental status of the mice across all groups. We further validated the hepato- and nephrotoxicity associated with the overdose of OM T-R Hydrogel in mice by analyzing serum biomarkers, with results presented in Figure 3. When the mice were administered an overdose of OM T - R Hydrogel, the serum levels of ALT, AST, and CREA in the OM T - R Hydrogel group were all significantly increased (*p < 0.05*). Additionally, the levels of ALP, BUN, and TBIL also showed an upward trend. These findings strongly indicate that an overdose of OM T-R Hydrogel can lead to liver and kidney damage. In the group that received an overdose of T-R Hydrogel, these indicators were elevated to varying degrees. The macroscopic examination of the isolated rectum revealed no signs of bleeding, necrosis, or other lesions. Histologically (Figure 4), the rectum exhibited elongated and straight intestinal glands, a significant presence of goblet cells, and a clearly defined structure across all layers, with no apparent lesions. Furthermore, there were no significant differences in histomorphology observed among the three groups.

## 4 Discussion

The main mode of OM absorption in the rectum is through the rectal veins. Drugs absorbed via the superior rectal veins undergo hepatic first-pass metabolism, while those absorbed via the middle or inferior rectal veins enter the systemic circulation directly through the inferior vena cava, which can effectively avoid the first-pass effect (27). The results of this study demonstrate that T-R Hydrogel serves as an effective drug carrier for the transrectal delivery of OM. In the pharmacokinetic analysis, it was observed that rectal administration of OM T-R Hydrogel does not induce a rapid elevation in systemic OM levels; instead, it results in a gradual increase in blood concentrations. At 50 min, the mean blood concentration in group R exhibited a relatively smooth decline after reaching its peak. Additionally, t_₁/₂λz_ was longer in group R compared to group O, indicating that OM T-R Hydrogel has the capacity for sustained drug release. These results indicate that rectal administration of OM T-R Hydrogel extends the dosing interval and decreases dosing frequency. Meanwhile, an analysis of T_max_ and C_max_ values for individual rabbits in Group R revealed substantial variability in drug absorption. Previous research has demonstrated that variable drug absorption remains one of the primary limitations of rectal drug delivery (27). In Group R rabbits, substantial variability was observed in C_max_ and T_max_, likely attributed to inter-individual metabolic disparities and feces. Rabbits were fasted for 16 h, and X-ray examination was performed to assess fecal content in the intestinal tract before the animal experiment. The cecum of rabbits is extremely well-developed, and they defecate frequently with no fixed timing (28). Therefore, during the experiment, we can only ensure that there is no feces in the colon and rectum segments. Unfortunately, this still cannot completely eliminate the interference of feces on the drug absorption. However, the defecation time of dogs and cats is relatively fixed, and there is less interference from feces in rectal management.

Most notably, drug accumulation can raise safety concerns associated with long-term drug use (29). Relevant studies indicate that prolonged use of OM does not result in significant liver or kidney damage (30). A study by Lopes et al. demonstrated that when cats received oral administration of OM at a dosage of 2 mg/kg for 28 consecutive days, only a small number of cats exhibited vomiting and diarrhea, and these symptoms cannot be directly attributed to OM (30). However, our findings from the acute toxicity test of OM T-R Hydrogel indicate that an overdose leads to hepatorenal injury, a result that is consistent with the effects observed from an overdose of T-R Hydrogel. These results indicated that overdosage of T-R Hydrogel leads to its accumulation in the body, which ultimately manifests as hepatorenal toxicity. HPMC, HP-*β*-CD, P407, and P188 are all materials with excellent biocompatibility, and no significant toxic effects are anticipated when used at low dosages (31–34). Therefore, it is sufficiently safe to use OM T-R Hydrogel according to the recommended dose.

The primary limitation of this study is the absence of an efficacy assessment to establish the effective blood concentration of rectally delivered drugs. Previous studies have shown that the C_max_ of oral OM (1 mg/kg) in cats is 1631.6 ng/mL (26). However, in this study, the C_max_ in rabbits after oral administration of 1 mg/kg OM was only 93.7 ng/mL. This indicates that under the same administration route and dose of OM, there is a significant difference in C_max_ among different species, which is related to species-specific variations in drug bioavailability (35, 36). Currently, the effective blood concentration of OM remains unknown. Oral administration of 0.4 mg/kg OM in dogs has been confirmed effective by the FDA, with a C_max_ of 259 ng/mL. In this study, the C_max_ of OM T-R Hydrogel following rectal administration in rabbits was 36.9 ng/mL. Assuming that the efficacy of OM across different animal species depends on achieving or maintaining the drug’s blood concentration, increasing the administered dose would be necessary to achieve therapeutic effects in rabbits. To further validate the effective blood concentration of OM T-R Hydrogel in rabbits, it is necessary to establish an itching model to assess therapeutic efficacy and determine the effective blood concentration.

Notably, this exploratory study only conducted relevant trials in rabbits, ultimately confirming that OM can effectively enter the systemic circulation following rectal administration of OM T-R Hydrogel. The clinical efficacy of OM T-R Hydrogel in dogs and cats needs to be definitively established through pharmacokinetic studies and efficacy evaluations in these species.

## 5 Conclusion

In this study, T-R Hydrogel was used as a drug delivery system for the transrectal administration of OM, resulting in a gradual increase in the plasma concentration of OM in rabbits. In conclusion, this exploratory study suggests that OM T-R Hydrogel may offer a novel therapeutic approach for pruritus management in dogs and cats. Future studies are also needed to evaluate its safety, optimal dosing, and therapeutic effectiveness across different breeds and severity levels of pruritus.

## Data Availability

The original contributions presented in the study are included in the article/Supplementary material, further inquiries can be directed to the corresponding authors.

## Supplementary material

Supplementary Table S1 The gelation behavior of T-R Hydrogel at 37°C and 38°C.
