# Supplementary material for: Temperature-responsive hydrogel of oclacitinib maleate administered via the rectum in rabbit
Source: Front Vet Sci. 2026 Feb 11;13:1767033. doi: 10.3389/fvets.2026.1767033 (PMC12932137; doi:10.3389/fvets.2026.1767033)
Supplement: Supplementary Table S1 — The gelation behavior of T-R Hydrogel at 37°C and 38°C. [file Table_1.docx]

The gelation behavior of T-R Hydrogel at 37°C

|  | 10 s | 20 s | 30 s | 33 s | 36 s | 39 s | 42 s | 45 s |
| --- | --- | --- | --- | --- | --- | --- | --- | --- |
| OM T-R Hydrogel | - | - | - | - | - | + | + | + |
| T-R Hydrogel | - | - | - | ＋ | + | + | + | + |

"-" denotes non-gelation，"+" denotes gelation (the same below).

The gelation behavior of T-R Hydrogel at 38°C

|  | 10 s | 20 s | 30 s | 33 s | 36 s | 39 s | 42 s | 45 s |
| --- | --- | --- | --- | --- | --- | --- | --- | --- |
| OM T-R Hydrogel | - | - | - | - | - | + | + | + |
| T-R Hydrogel | - | - | + | + | + | + | + | + |
